# Synchronous Pulmonary Squamous Cell Carcinoma and Mantle Cell Lymphoma of the Lymph Node

**DOI:** 10.1155/2011/945181

**Published:** 2011-07-02

**Authors:** Yu Sun, Yun-Fei Shi, Li-Xin Zhou, Ke-Neng Chen, Xiang-Hong Li

**Affiliations:** ^1^Department of Pathology, Key Laboratory of Carcinogenesis and Translational Research (Ministry of Education), Beijing Cancer Hospital & Institute, Peking University School of Oncology, NO. 52 Fu-Cheng Road, HaiDian District, Beijing 100142, China; ^2^Department of Thoracic Surgery, Key Laboratory of Carcinogenesis and Translational Research (Ministry of Education), Beijing Cancer Hospital & Institute, Peking University School of Oncology, Beijing, China

## Abstract

Synchronous occurrence of pulmonary squamous cell carcinoma and malignant lymphoma of the lymph node is not reported in the literature. We report a case of pulmonary squamous cell carcinoma coexisting with a mantle cell lymphoma involving cervical and mediastinal lymph node. It is important to recognize this synchronous occurrence histopathologically and to be aware of the existence of “in situ” MCL.

## 1. Introduction

Recent epidemiologic evidence suggests that lung cancer is the leading cause of cancer mortality in both sexes [1]. Squamous cell carcinoma (SCC) is a malignant epithelial tumor characterized by the presence of keratinization and intercellular bridges. It is graded according to the degree of keratinization, squamous pearl formation, or intercellular bridges. These features are obvious in the well-differentiated tumors but only focally demonstrated in the poorly differentiated tumors.

Mantle cell lymphoma (MCL) is a B-cell neoplasm generally composed of monomorphic small to medium-sized lymphoid cells, characterized by t(11;14)(q13;q32) and Cyclin D1 overexpression, comprising from 3% to 10% of all non-Hodgkin's lymphomas [2, 3]. The affected patients are mainly middle-aged or older, and they are often presented with Stages III or IV disease [4].

Synchronous occurrence of pulmonary squamous cell carcinoma and malignant lymphoma of the lymph node is not reported until today, and we report a unique case of a pulmonary squamous cell carcinoma coexisting with a mantle cell lymphoma involving cervical and mediastinal lymph node.

## 2. Case Report

**Figure 1:**
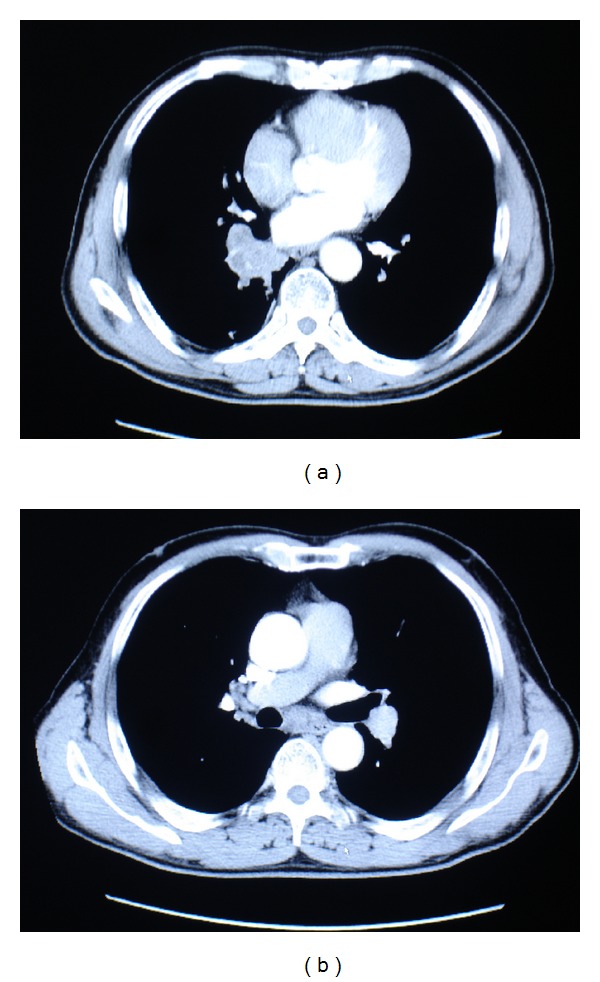
Preoperative chest CT scan revealed a mass in the right lower lobe (a), with enlargement of mediastinal lymph nodes (b).

A 57-year-old male patient was admitted to our clinic with a five-month history of multiple bilateral nodules in the neck. He had cough, expectoration, and low fever in the previous one month. A thoracic-computed tomographic (CT) examination revealed a 4.5 × 3.0 cm mass in the right lower lobe, with enlargement of multiple mediastinal lymph nodes (Figure 1). 

Subsequently, he underwent CT-guided fine needle biopsy of the right lower lobe and biopsy of the right cervical lymph node. Microscopic examination revealed poorly differentiated squamous cell carcinoma of the right lung and mantle cell lymphoma of the right cervical lymph node. The patient received 2 cycles of preoperative chemotherapy. Subsequently, he underwent a lower lobectomy of the right lung and received 4 cycles of postoperative chemotherapy.

## 3. Materials and Methods

After fixation in 10% buffered formalin, tumors were sampled according to standard procedures and processed into paraffin embedding. Serial 4 **μ**m thick sections were cut and stained with haematoxylin-eosin (H&E) for conventional histology.

Immunohistochemical staining was conducted with Ventana Automated immunohistochemistry instrument. Primary antibodies included CK5/6, P63, CK8/18, LCA, CD20, CD5, Cyclin D1, Bcl-2, Bcl-6, CD3, CD10, CD21, CD23, and Ki-67 applied at appropriate dilutions. Sections known to express high levels of all primary antibodies were included as positive controls, while negative control slides omitted the primary antibody. 

Interphase FISH analysis was also performed using CCND1 Dual color, break apart rearrangement probe, and IGH/CCND1 Dual color, dual fusion translocation probe (all from Vysis/Abbott Ltd, USA).

## 4. Results

### 4.1. Pathological Findings and Immunohistochemistry

**Figure 2:**
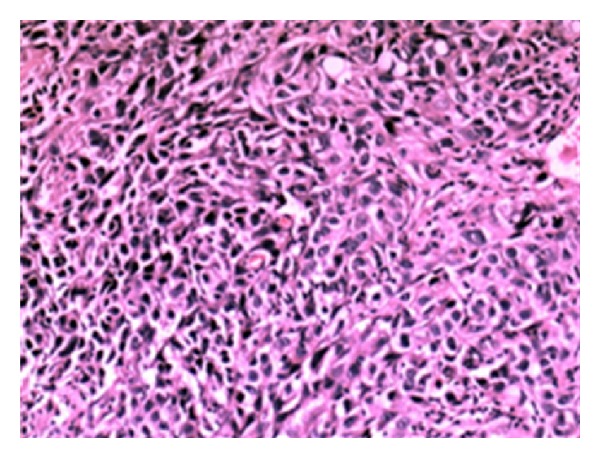
Histopathological examination revealed squamous cell carcinoma of the lung.

Microscopic examination of fine needle biopsy revealed a malignant epithelial tumor composed of atypical cells with large vesiculated nuclei and scant cytoplasm. The histopathological diagnosis was “poorly differentiated squamous cell carcinoma” (Figure 2). Immunohistochemical stains showed the following tumor-cell immunophenotype: CK5/6(+), P63(+), CK8/18(−), and LCA(−).

**Figure 3:**
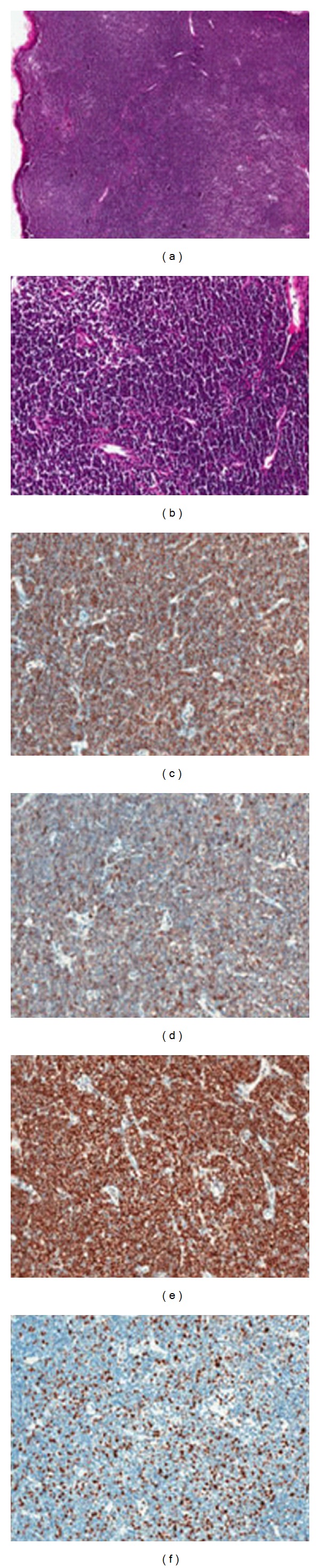
Mantle cell lymphoma with diffuse growth pattern of the cervical lymph node (a, b). Neoplastic cells, positive for CD20 (c), CD5 (d), and cyclin D1 (e). Ki-67 label index about 20–30% (f).

**Figure 4:**
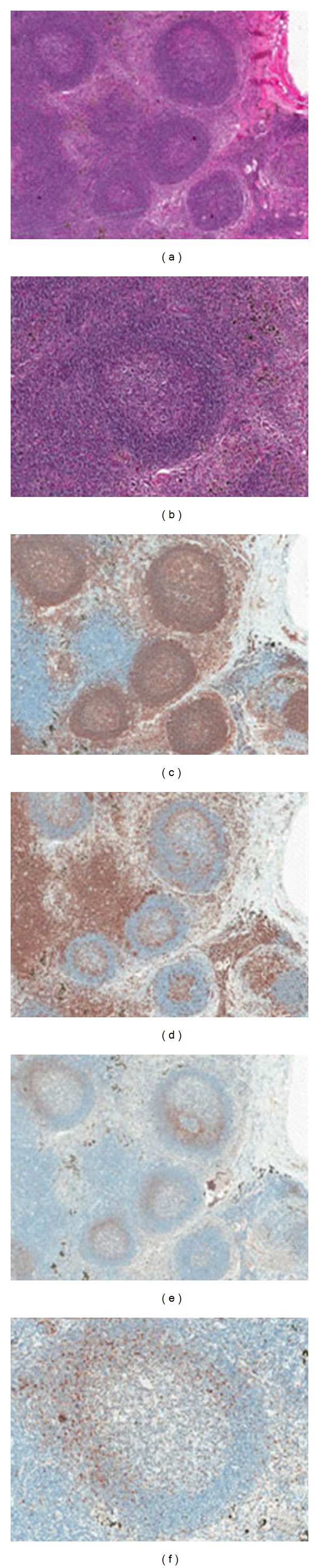
“in situ” MCL of the mediastinal lymph node (a, b). The follicles were strongly positive for CD20 (c). Neoplastic cells were negative for CD3 (d). Neoplastic cells were positive for cyclin D1 and presented a focal colonization of the mantle zones of lymphoid follicles (e, f).

Biopsy of the right cervical lymph node revealed mantle cell lymphoma (Figure 3). The lymphoid cells were medium-sized, with rounded or angular nuclei and with one or more indistinct nucleoli. Focally, there was a nodular pattern of growth. The immunophenotype of the lymphoma cells was the following: CD20(+), CD5(+), CyclinD1(+), Bcl-2(+), Bcl-6(−), CD3(−), CD10(−), CD21(−), CD23(−), Ki-67(+20–30%) (Figure 4).

Histopathological findings of lobectomy confirmed the diagnosis of poorly differentiated SCC, with degeneration of tumor cell and abundant necrosis. There were also mediastinal lymph node metastases. Many lymph nodes showed prominent and heterogeneous follicular hyperplasia with no diffuse or confluent areas. Irregularly shaped follicles were tightly packed, but the interfollicular zones were not infiltrated. Germinal centres were preserved and surrounded by a normal looking or mildly enlarged, excentric mantle zone. The mantle zone showed a monotonous population of small to medium-sized cells, with slightly indented and irregular nuclei, inconspicuous nucleoli, and scant cytoplasm. The interfollicular zones were filled with a mixed cell population, but no atypical cell was seen. The follicles were strongly positive for CD20. Few B cells were present in interfollicular zones. There was focal colonization of tumor B-cells expressing cyclinD1 in the mantle zone areas of lymphoid follicles, which was diagnosed as early phase of mantle cell lymphoma. We found no cyclin D1 expression in the interfollicular areas. Neoplastic cells were negative for CD3. These data and the morphological preservation of the mantle zones prompted us to name this lesion in situ MCL.

### 4.2. Fluorescence In Situ Hybridization (FISH)

**Figure 5:**
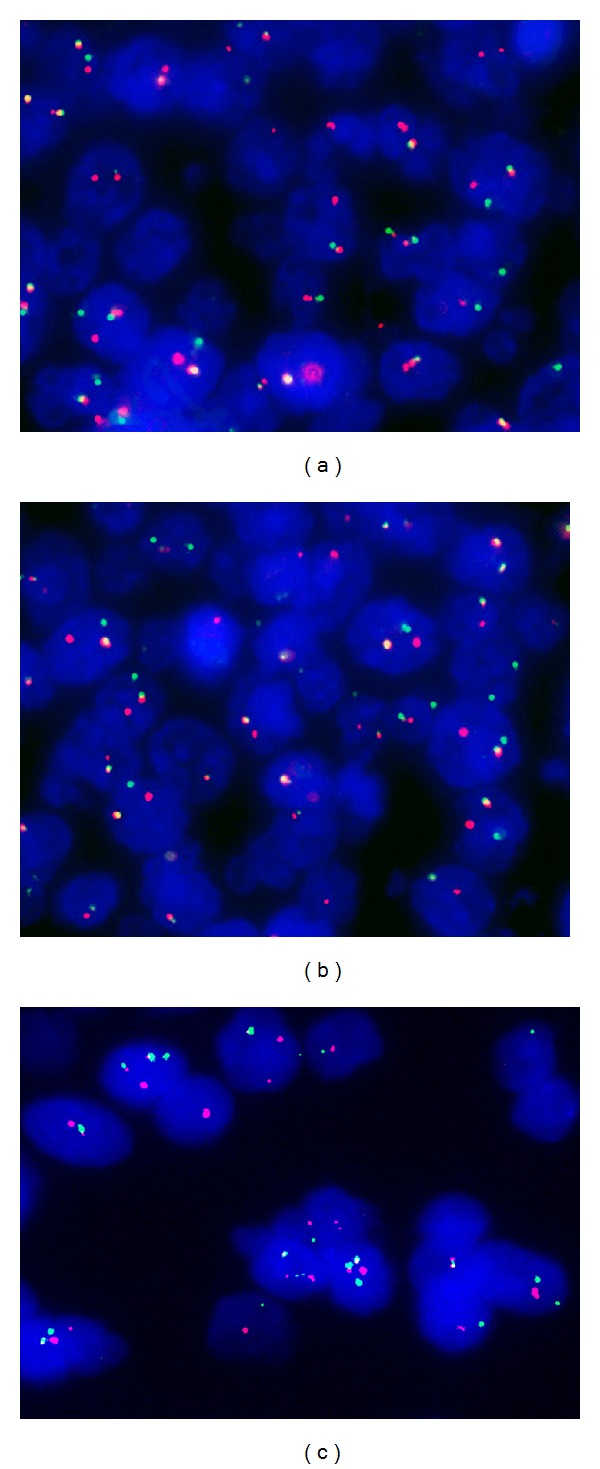
FISH method using the CCND1 Dual color, break apart rearrangement probe: nucleus of neoplastic lymphoid cell with t(11;14) displaying the signal pattern 1 yellow (fusion translocation signals), 1 red (CCND1 gene), and 1 green (IGH gene) ((a) for CCND1 gene break and (b) for IGH gene break). FISH method using the IGH/CCND1 dual color, dual fusion translocation probe: nucleus of neoplastic lymphoid cell with t(11;14) displaying the signal pattern 2 yellow (fusion translocation signals), 1 red (CCND1 gene), and 1 green (IGH gene) (c).

The characteristic translocation between CCND1 gene on chromosome 11 and IGH gene on chromosome 14 [t(11;14)] was found in both cervical and mediastinal lymph nodes (Figure 5). The FISH results showed two distinctive signals and one fused signal in each tumor cell, thus indicating the translocation of IgH and Cyclin D1 loci and confirming that both cervical and mediastinal lymph node were MCL.

### 4.3. Treatment

The patient received 2 cycles of preoperative chemotherapy (Paclitaxe and Cisplatin). Subsequently, he underwent a lower lobectomy of the right lung and received 4 cycles of postoperative chemotherapy (Gemzar and Cisplatin).

### 4.4. Clinical Followup

The patient's clinical status at 23 months after the diagnosis is good, without any evidence of local recurrence, metastatic disease, or lymph node involvement, according to the followup CT scan imaging of thorax, abdomen, and brain.

## 5. Discussion

Synchronous occurrence of pulmonary SCC and MCL of lymph node is not reported in the medical literature up to now. Extensive search of the literature revealed few cases with coexistence of different types of lung carcinomas and malignant lymphomas. Chanel et al. described a synchronous pulmonary adenocarcinoma and extranodal marginal zone lymphoma of MALT type [5]. Rothenburger et al. reported a non-Hodgkin's lymphoma coexisting with an NSCLC, whereas Rubiales et al. described the synchronous occurrence of a small-cell lung cancer and a Hodgkin lymphoma [6, 7]. 

The histologic pattern of MCL may be diffuse, nodular, or mantle zone or a combination of the three patterns of growth. Infrequent cases may show involvement almost exclusively restricted to the inner mantle zones or to narrow mantles (“in situ” MCL) [8, 9]. Mantle zone pattern and in situ MCL are different concepts. The mantle zone pattern refers to the expansion of the mantle zone by MCL with preservation of small or hyperplastic germinal centers and overall preservation of the architecture. In situ MCL refers to the focal colonization of the mantle zones by MCL cells. The case reported in our study presented a diffuse growth pattern in the cervical lymph node and a focal colonization of the mantle zones by MCL cells which we designated in situ in the mediastinal lymph nodes. Diagnosis may be difficult because the lymph node architecture is preserved. Cytologically, however, monotony in lymphoid cell size and irregularity of nuclear shape suggested the need for immunohistochemical study. 

Cyclin D1 staining may be required to recognize the cases for “in situ” MCL. As cyclin D1 is not found in appreciable amounts in reactive lymphadenitis (only nuclei of reactive macrophages and stromal cells are consistently positive), this marker was of utmost importance in the diagnosis of MCL. In this case, there was a focal colonization of tumor B-cells expressing cyclinD1 in the mantle zone areas of lymphoid follicles. Meanwhile, interphase FISH analysis also revealed the characteristic translocation between CCND1 gene and IGH gene. These data and the morphological preservation of the mantle zones prompted us to name this lesion in situ MCL. 

From this case, we want to suggest that for a pathologist, it is crucial to recognize this synchronous occurrence histopathologically. While looking at the lymph nodes from a resection specimen with a known carcinoma, pathologists tend to focus on picking up a metastatic lesion rather than to look at the background architecture of the lymph node. Any effacement of the architecture needs a closer inspection to exclude the possibility of an underlying lymphoma, as it usually has a bearing on both further treatment and prognosis. On the other hand, the main information obtained from this study is that pathologists should be aware of the existence of “in situ” MCL, and demand an adequate immunohistochemical panel, including a marker for cyclinD1, to differentiate this neoplasm from follicular hyperplasia and other lymphoma with pseudofollicular patterns.
